# Recurrent symphysitis culminating in pelvic ring fracture after hyperextended transurethral prostate resection and vaporization with symphysis erosion: a case report

**DOI:** 10.1186/s13256-017-1292-5

**Published:** 2017-05-19

**Authors:** Holger Gerullis, Arne Eitzen, Jens Uphoff, Fadi Daaboul, Ajay Chavan, Leander Ermert, Friedhelm Wawroschek, Alexander Winter

**Affiliations:** 1 School of Medicine and Health Sciences, University Hospital for Urology, Klinikum Oldenburg, Carl von Ossietzky University Oldenburg, Rahel-Straus-Straße 10, 26133 Oldenburg, Germany; 2 Department of Diagnostic and Interventional Radiology, Klinikum Oldenburg, Oldenburg, Germany; 3 Oldenburg Institute of Pathology, Oldenburg, Germany

**Keywords:** Pelvic ring fracture, Prostate resection, Complication, TURP, TUVP

## Abstract

**Background:**

Short-term and long-term complications of transurethral prostate resection can be different in nature. Capsule perforation and subsequent fistulation after resection and electrovaporization is seldom reported in the literature.

**Case presentation:**

Here we report the case of a 79-year-old caucasian man with capsule perforation after transurethral prostate resection and electrovaporization resulting in a severe and recurrent symphysitis and subsequent pelvic ring fracture. The bladder-symphysis fistulation was surgically removed and additional orthopedic surgery could be avoided after definitely solving the urological problem.

**Conclusions:**

Urologists should be aware of rare complications after transurethral resection and electrovaporization of the prostate.

## Background

As recently analyzed by Gilfrich and colleagues on the basis of over 95,000 cases, transurethral resection of the prostate (TURP) represents the most common surgical treatment approach for lower urinary tract symptoms (LUTS) in Germany [1]. Yet, the proportion of its use has decreased during recent years because of the increasing use of laser technique-based procedures [1]. Side effects and complications of TURP are repeatedly reported in the literature. Perioperative complications associated with TURP are known to differ depending on the usage of monopolar or bipolar techniques [2].

Bleeding, consecutive blood transfusions, and transurethral resection (TUR) syndrome are the most relevant intraoperative complications [3–5]. Complications of the short-term and midterm follow-up are urethral stricture [6], bladder neck obstruction, or recurrent adenoma [7]. Perforation of the capsule of the prostate has been described rarely [8]. However, those perforations have seldom led to significant long-term complications.

In this case report we describe a patient with recurrent symphysitis culminating in pelvic ring fracture as a midterm complication following hyperextended prostate and capsule resection and electrovaporization with symphysis erosion as a very rare and not yet reported complication of transurethral surgical cure of benign prostate hyperplasia.

## Case presentation

A 79-year-old white man (born in 1937) presented for the first time at our urology department in June 2016. He had several relevant comorbidities: chronic obstructive pulmonary disease, infrarenal aneurysm of the abdominal aorta, status post-sigma resection due to acute diverticulitis in 2011, obesity, peripheral vascular disease stage IIa, arteriosclerosis with occlusion of left external iliac artery, several allergies to antibiotics, arterial hypertension, left heart insufficiency, and hyperlipidemia.

He was initially admitted due to acute suprapubic pain on the basis of an acute symphysitis. He had several previous urologic surgeries which are displayed in chronological order in Table 1.

**Table 1:** Surgical interventions related to the current medical problem in patient’s medical records

Nr	Date	Indication	Procedure	Findings	Complications	Comments
1	10/2011	Subvesical obstruction	TUR-prostate (performed in external institution)	Resection of 29 g benign prostate adenoma tissue	None	Operation time: 45 min Sufficient functional results in the short-term follow-up. Primary recovery
2	02/2016	1. Recurrent subvesical obstruction 2. Suspicious bladder formation left bladder wall	1. Transurethral electrovaporization and resection of the prostate 2. Resection of suspicious bladder formation (both procedures performed in external institution)	1. Obstruction cleared by resection and vaporization of 18 g residual adenoma (sonography), resected weight 4 g 2. Histologic exclusion of bladder cancer	Postoperative urinary retention and subsequent insertion of suprapubic catheter (day 5 postoperation)	Operation time: 55 min Primary recovery, suspicious resected bladder formation not close to the bladder neck/anterior bladder wall
3	06/2016	Suspicion of necrotic area and possible carcinoma formation in prostate capsule	Diagnostic urethrocystoscopy, cystography, transurethral resection of the ventral part of the prostate capsule	Detection of an erosion in ventral part of resected prostate, exclusion of mesh erosion, necrotic tissue, histological exclusion of carcinoma, detection of tissue of an osseous nature at the base of erosion zone	None	Primary recovery
4	08/2016	Abscess formation	Incision and debridement of a right-side inguinal/periscrotal abscess	Abscess formation		Primary recovery
5	10/2016	Fistula prostate capsule/symphysis	Open excision of prostate fistula and coverage with bladder flap	Fistulation		Primary recovery, no recurrent fistula during 6 months of follow-up

*TUR* transurethral resection

**Fig. 1:**
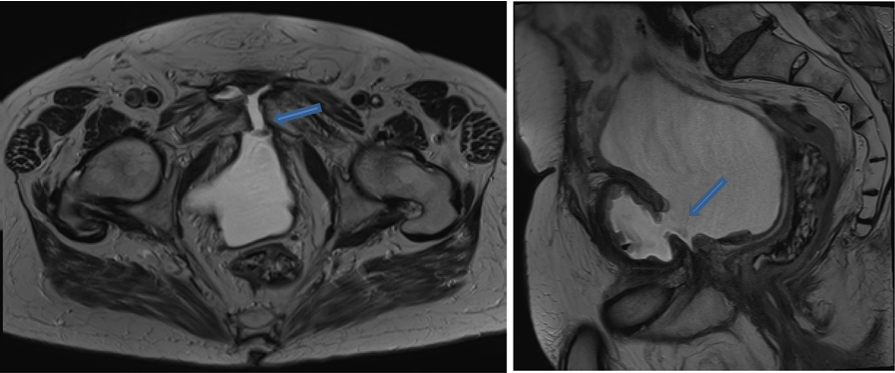
Fistulation between urinary bladder and fluid collection of the symphysis (*arrows*)

**Fig. 2:**
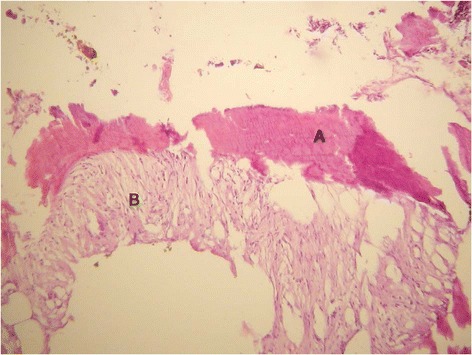
Microscopic impression of resected specimen during surgery (Number 3 of Table 1). **a** represents bone, **b** represents connective tissue, probably originating from the prostate capsule or residual adenoma

A magnetic resonance imaging (MRI) revealed a fistulation between his bladder and a collection in the lower abdominal wall attached to the symphysis with an extended inflammatory reaction of all involved osseous structures and soft tissue (Fig. 1). Endoscopic evaluation did then show an excessively resected prostate capsule and an erosion of the ventral part of the capsule. Resection in this area revealed osseous structure which was histologically confirmed (Fig. 2). His postoperative recovery was slow. In August 2016 an inguinal/periscrotal abscess formation had to be treated surgically. This abscess was most probably related to the persisting fistulation resulting from the previous surgeries.

**Fig. 3:**
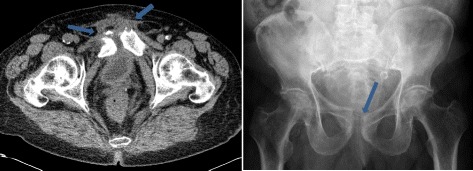
Symphysitis (computed tomography scan, *left*) and dilatation of the symphysis fissure and osseous erosions (conventional radiography, *right*) (*arrows*)

**Fig. 4:**
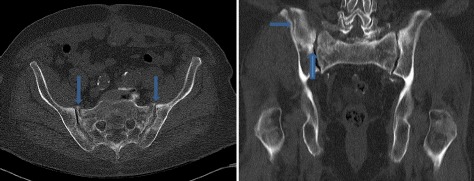
Fracture of the right iliac crest and pelvic ring fracture (computed tomography scan) (*arrows*)

As his mobility steadily decreased and he had massive pelvic pain in the following weeks, a ring fracture of the pelvis, most probably on the basis of the destabilizing recurrent symphysitis, was diagnosed radiologically (Figs. 3 and 4).

In October 2016 we explored his suprapubic region surgically. The fistulation was detected in the transition zone from bladder neck and prostate capsule as expected. In order to definitely cure the fistulation of the prostate capsule and symphysis we performed a total fistula excision and coverage with bladder flap. In the postoperative course he recovered quickly and was discharged 12 days after the surgery. He is regularly seen and conservatively treated in our orthopedic department and he did not have to undergo further surgeries.

## Discussion

Over the last decades TURP has been the primary method to relieve bladder outlet obstruction for patients with benign disease. The procedure has been improved over the years resulting in significant decreases of mortality and morbidity. A gland larger than 45 g, an operative time longer than 90 minutes, and surgery after acute urinary retention are seen as risk factors for increased postoperative morbidity [9]. In a large-scale prospective multicenter evaluation in Germany comprising 44 mostly non-academic urological departments, Reich and colleagues confirmed that morbidity and mortality rates of TURP were closely related to the resection weight [4]. The patient in this case had a primary resection weight of 29 g, thus a considerably small value. However, nothing is known about the learning curve position of both the primary and the secondary surgeon. In addition, his second transurethral procedure, 5 years later in February 2016 revealed a residual adenoma of an additional 18 g (transrectal ultrasound), which was located predominantly in the apical region. Analyzing over 20,000 men from Austria, Madersbacher and co-workers reported a secondary surgical procedure after initial TURP necessary due to functional insufficiency of 5.8%, 12.3%, and 14.7% at 1, 5, and 8 years of follow-up, respectively [10]. This is in compliance with the case reported here. In fact, the risk of perioperative complications increases with the number of surgeries.

There is a high index of suspicion that the compromising damage to the capsule was produced during the secondary surgery because the patient’s pain increased and signs of symphysitis occurred after this second procedure.

Transurethral vaporization of the prostate (TUVP) as performed in this patient has been investigated *in vitro* by Reich *et al*. [11]. They reported a 15 to 20 times higher energy demand to be considered when applying the vaporization technique in order to achieve comparable results to conventional TURP. In addition, the authors found a remarkable decline in tissue removal of more than 50% for TUVP on pretreated tissue, which can only be compensated *in vivo* by an additional energy input. Compared to standard TURP, pure electrovaporization as done in this particular patient requires slower speed, higher voltage, and increased pressure application [11]. In this case, the second surgery in February 2016 was of 55 minutes’ duration, which was 10 minutes longer than the initial TURP in 2011. Thus, a high energy level over a considerably longer surgical time on a preoperated prostate can be assumed and is very likely the reason for the capsule destruction. Combination of monopolar resection and vaporization may have further increased the risk of wall rupture. Since TUVP took place in another institution no data were available regarding the respective energy settings, although those settings, if extended, might represent an additional negative factor.

Pubic symphysitis has been reported as a rare complication after TURP by Ziesel *et al*. who found 12 cases among 12,118 patients having undergone TURP over 15 years [12]. The authors could not find a single cause for developing symphysitis but remarked an overrepresentation of suprapubic trocar placement, chronic prostatic inflammation, and extended resection. In addition, they concluded that inflammatory, thermic, and/or surgical damage of the capsule were likely to be causative which is in accordance with the case reported here.

All of the above-mentioned factors combined may easily explain how the capsule perforation occurred in this patient and led to the described subsequent complications.

In the subsequent follow-up it took several months to finally detect the cause of the symphysitis, fistula development, and resulting pelvic instability and ring fracture. The definitive diagnosis was completed by another endoscopic procedure. The surgical treatment approach led to definitive fistula resection and is experimental in nature.

The performed patient-tailored innovative surgical approach led to significantly decreased pain and improved the quality of life (QoL) of the patient. The pelvic ring fracture could be treated conservatively in the short-term follow-up of 6 months.

## Conclusions

This is to the best of our knowledge the first case of a symphysitis after capsule perforation during transurethral ablative surgeries leading to instability of the complete pelvis. Urologists need to be aware of this rare complication after transurethral procedures.

## Abbreviations

LUTS: Lower urinary tract symptoms
QoL: Quality of life
TUR: Transurethral resection
TURP: Transurethral resection of the prostate
TUVP: Transurethral vaporization of the prostate
